# Supplementary material for: Promising Effects of Digital Chest Tube Drainage System for Pulmonary Resection: A Systematic Review and Network Meta-Analysis
Source: J Pers Med. 2022 Mar 22;12(4):512. doi: 10.3390/jpm12040512 (PMC9029690; doi:10.3390/jpm12040512)
Supplement: Supplementary file 1 [file jpm-12-00512-s001.zip › jpm-1635162-supplementary.pdf]

Supplementary Table

Promising Effects of Digital Chest Tube Drainage System for Pulmonary Resection: A Systematic Review and Network Meta-Analysis

Po-Chih Chang[1,2,3,4], Kai-Hua Chen[5#], Hong-Jie Jhou[6], Cho-Hao Lee[7], Shah-Hwa Chou[1,8], Po-Huang Chen[9+], Ting-Wei Chang[5*]

Contents

Supplementary Table S1. PRISMA for Network Meta-Analyses

Supplementary Table S2. Search strategy

Supplementary Table S3. Characteristics of included studies

Supplementary Table S4. Results the head-to-head comparison of network meta-analysis

Supplementary Table S5. Estimation of inconsistency

Supplementary Table S6. Risk of bias table of included studies

Supplementary Table S7. Sensitivity analyses

Supplementary Table S8. Reference list of excluded studies

Supplementary Table S9. Reference list of included studies

Supplementary Table S1. PRISMA for Network Meta-Analyses

Section/ Topic	Item	Checklist Item	Reported on Page #
**TITLE**			
Title	1	Identify the report as a systematic review *incorporating a network meta-analysis (or related form of meta-analysis).*	Title page
**ABSTRACT**			
Structured summary	2	Provide a structured summary including, as applicable:<br>**Background:** main objectives<br>**Methods:** data sources; study eligibility criteria, participants, and interventions; study appraisal; and *synthesis methods, such as network meta-analysis.*<br>**Results:** number of studies and participants identified; summary estimates with corresponding confidence/credible intervals; *treatment rankings may also be discussed. Authors may choose to summarize pairwise comparisons against a chosen treatment included in their analyses for brevity.*<br>**Discussion/Conclusions:** limitations; conclusions and implications of findings.<br>**Other:** primary source of funding; systematic review registration number with registry name.	1
**INTRODUCTION**			
Rationale	3	Describe the rationale for the review in the context of what is already known, *including mention of why a network meta-analysis has been conducted.*	2, 3
Objectives	4	Provide an explicit statement of questions being addressed, with reference to participants, interventions, comparisons, outcomes, and study design (PICOS).	3
**METHODS**			
Protocol and registration	5	Indicate whether a review protocol exists and if and where it can be accessed (e.g., Web address); and, if available, provide registration information, including registration number.	4
Eligibility criteria	6	Specify study characteristics (e.g., PICOS, length of follow-up) and report characteristics (e.g., years considered, language, publication status) used as criteria for eligibility, giving rationale. *Clearly describe eligible treatments included in the treatment network, and note whether any have been clustered or merged into the same node (with justification).*	5
Information sources	7	Describe all information sources (e.g., databases with dates of coverage, contact with study authors to identify additional studies) in the search and date last searched.	4,5

Search	8	Present full electronic search strategy for at least one database, including any limits used, such that it could be repeated.	4
Study selection	9	State the process for selecting studies (i.e., screening, eligibility, included in systematic review, and, if applicable, included in the meta-analysis).	5
Data collection process	10	Describe method of data extraction from reports (e.g., piloted forms, independently, in duplicate) and any processes for obtaining and confirming data from investigators.	5, 6
Data items	11	List and define all variables for which data were sought (e.g., PICOS, funding sources) and any assumptions and simplifications made.	6,7
**Geometry of the network**	**S1**	Describe methods used to explore the geometry of the treatment network under study and potential biases related to it. This should include how the evidence base has been graphically summarized for presentation, and what characteristics were compiled and used to describe the evidence base to readers.	7,8
Risk of bias within individual studies	12	Describe methods used for assessing risk of bias of individual studies (including specification of whether this was done at the study or outcome level), and how this information is to be used in any data synthesis.	9
Summary measures	13	State the principal summary measures (e.g., risk ratio, difference in means). *Also describe the use of additional summary measures assessed, such as treatment rankings and surface under the cumulative ranking curve (SUCRA) values, as well as modified approaches used to present summary findings from meta-analyses.*	7,8
Planned methods of analysis	14	Describe the methods of handling data and combining results of studies for each network meta-analysis. This should include, but not be limited to:<br>*Handling of multi-arm trials;*<br>*Selection of variance structure;*<br>*Selection of prior distributions in Bayesian analyses; and Assessment of model fit.*	6 - 9
**Assessment of Inconsistency**	**S2**	Describe the statistical methods used to evaluate the agreement of direct and indirect evidence in the treatment network(s) studied. Describe efforts taken to address its presence when found.	8,9
Risk of bias across studies	15	Specify any assessment of risk of bias that may affect the cumulative evidence (e.g., publication bias, selective reporting within studies).	9
Additional analyses	16	Describe methods of additional analyses if done, indicating which were pre-specified. This may include, but not be limited to, the following:<br>Sensitivity or subgroup analyses;<br>Meta-regression analyses;<br>*Alternative formulations of the treatment network; and Use of alternative prior distributions for Bayesian analyses (if applicable).*	9

**RESULTS**			
Study selection	17	Give numbers of studies screened, assessed for eligibility, and included in the review, with reasons for exclusions at each stage, ideally with a flow diagram.	10, Figure 1
**Presentation of network structure**	**S3**	Provide a network graph of the included studies to enable visualization of the geometry of the treatment network.	Figure 2
**Summary of network geometry**	**S4**	Provide a brief overview of characteristics of the treatment network. This may include commentary on the abundance of trials and randomized patients for the different interventions and pairwise comparisons in the network, gaps of evidence in the treatment network, and potential biases reflected by the network structure.	eFigure 3
Study characteristics	18	For each study, present characteristics for which data were extracted (e.g., study size, PICOS, follow-up period) and provide the citations.	10,11, Table1, eTable 3
Risk of bias within studies	19	Present data on risk of bias of each study and, if available, any outcome level assessment.	13, 14, eTable 6
Results of individual studies	20	For all outcomes considered (benefits or harms), present, for each study: 1) simple summary data for each intervention group, and 2) effect estimates and confidence intervals. *Modified approaches may be needed to deal with information from larger networks.*	11-13
Synthesis of results	21	Present results of each meta-analysis done, including confidence/credible intervals. *In larger networks, authors may focus on comparisons versus a particular comparator (e.g. placebo or standard care), with full findings presented in an appendix. League tables and forest plots may be considered to summarize pairwise comparisons.* If additional summary measures were explored (such as treatment rankings), these should also be presented.	11-13
**Exploration for inconsistency**	**S5**	Describe results from investigations of inconsistency. This may include such information as measures of model fit to compare consistency and inconsistency models, *P* values from statistical tests, or summary of inconsistency estimates from different parts of the treatment network.	12, 13
Risk of bias across studies	22	Present results of any assessment of risk of bias across studies for the evidence base being studied.	13, 14
Results of additional analyses	23	Give results of additional analyses, if done (e.g., sensitivity or subgroup analyses, meta-regression analyses*, alternative network geometries studied, alternative choice of prior distributions for Bayesian analyses,* and so forth).	13, 14
**DISCUSSION**			
Summary of evidence	24	Summarize the main findings, including the strength of evidence for each main outcome; consider their relevance	15

		to key groups (e.g., healthcare providers, users, and policy-makers).	
Limitations	25	Discuss limitations at study and outcome level (e.g., risk of bias), and at review level (e.g., incomplete retrieval of identified research, reporting bias). *Comment on the validity of the assumptions, such as transitivity and consistency. Comment on any concerns regarding network geometry (e.g., avoidance of certain comparisons).*	18, 19
Conclusions	26	Provide a general interpretation of the results in the context of other evidence, and implications for future research.	20
**FUNDING**			
Funding	27	Describe sources of funding for the systematic review and other support (e.g., supply of data); role of funders for the systematic review. This should also include information regarding whether funding has been received from manufacturers of treatments in the network and/or whether some of the authors are content experts with professional conflicts of interest that could affect use of treatments in the network.	21

PICOS = population, intervention, comparators, outcomes, study design.
* Text in italics indicateS wording specific to reporting of network meta-analyses that has been added to guidance from the PRISMA statement.

Supplementary Table S2. Search Strategy

Data base	Search Strategy
Pubmed	((("pulmonary surgical procedures"[MeSH Terms] OR ("pulmonary"[All Fields] AND "surgical"[All Fields] AND "procedures"[All Fields]) OR "pulmonary surgical procedures"[All Fields] OR ("lung"[All Fields] AND "surgery"[All Fields]) OR "lung surgery"[All Fields] OR (("lung"[MeSH Terms] OR "lung"[All Fields] OR "pulmonary"[All Fields]) AND ("resect"[All Fields] OR "resectability"[All Fields] OR "resectable"[All Fields] OR "resectates"[All Fields] OR "resected"[All Fields] OR "resecting"[All Fields] OR "resection"[All Fields] OR "resectional"[All Fields] OR "resectioned"[All Fields] OR "resectioning"[All Fields] OR "resections"[All Fields] OR "resective"[All Fields] OR "resects"[All Fields]))) AND ("chested"[All Fields] OR "thorax"[MeSH Terms] OR "thorax"[All Fields] OR "chest"[All Fields] OR "chests"[All Fields] OR ("lung"[MeSH Terms] OR "lung"[All Fields] OR "pulmonary"[All Fields]) OR ("pleura"[MeSH Terms] OR "pleura"[All Fields] OR "pleural"[All Fields]) OR ("digital"[All Fields] OR "digitalisation"[All Fields] OR "digitalised"[All Fields] OR "digitalization"[All Fields] OR "digitalize"[All Fields] OR "digitalized"[All Fields] OR "digitalizer"[All Fields] OR "digitalizing"[All Fields] OR "digitally"[All Fields] OR "digitals"[All Fields] OR "digitization"[All Fields] OR "digitizations"[All Fields] OR "digitize"[All Fields] OR "digitized"[All Fields] OR "digitizer"[All Fields] OR "digitizers"[All Fields] OR "digitizes"[All Fields] OR "digitizing"[All Fields]))) AND

	("drainage"[MeSH Terms] OR "drainage"[All Fields] OR "drainaged"[All Fields] OR "drainages"[All Fields] OR ("suction"[MeSH Terms] OR "suction"[All Fields] OR "suctioning"[All Fields] OR "suctions"[All Fields] OR "suctioned"[All Fields] OR "suctionings"[All Fields]) OR (("air"[MeSH Terms] OR "air"[All Fields]) AND "leak"[All Fields]))) AND (clinicaltrial[Filter] OR randomizedcontrolledtrial[Filter])
Embase, MEDLINE	('lung surgery'/exp OR 'lung surgery' OR (('lung'/exp OR lung) AND ('surgery'/exp OR surgery)) OR 'pulmonary resection'/exp OR 'pulmonary resection' OR (pulmonary AND ('resection'/exp OR resection))) AND ('chest'/exp OR chest OR pulmonary OR pleural OR digital) AND ('drainage'/exp OR drainage OR 'suction'/exp OR suction OR 'air leak'/exp OR 'air leak' OR (('air'/exp OR air) AND ('leak'/exp OR leak))) AND ([controlled clinical trial]/lim OR [randomized controlled trial]/lim)
Cochrane Library	Trials matching "#1 - ((lung surgery OR pulmonary resection)):ti,ab,kw AND ((chest OR pulmonary OR pleural or digital)):ti,ab,kw AND ((drainage OR suction OR air leak)):ti,ab,kw (Word variations have been searched)" in Trials (Word variations have been searched)

Supplementary Table S3. Characteristics of included studies.

Table. Characteristics of included studies

Study	Study Design	Nationality	Scenario	Intervention 1	Intervention 2
Marshall 2002	RCT	The US	NR	Traditional suction (-20 cm H_2O suction)	Water seal
Ayed 2003	RCT	Kuwait	Primary spontaneous pneumothorax :100%	Traditional suction (-20 cm H_2O suction)	Water seal
Brunelli 2004	RCT	Italy	Lung cancer: 100%	Traditional suction (-20 cm H_2O suction)	Water seal
Alphonsoa 2005	RCT	The UK	NR	Traditional wall-mounted suction (2 kPa.)	Water seal
Brunelli 2005	RCT	Italy	Lung cancer: 100%	Traditional suction (-10 cm H_2O suction)	Water seal
Kakhki 2006	RCT	Iran	NR	Traditional suction (-10/-16 to -18 cm H_2O suction)	Water seal
Cerfolio 2008	RCT	The US	Malignant pulmonary tumor: 85%<br>Benign pulmonary tumor: 15%	Digital drainage system: Digivent® (Millicore, Sweden)	Water Seal: Sahara S-11000® (Teleflex, Research Triangle Plus, NC, US)
Prokakis 2008	RCT	Greece	Lung cancer: 100%	Traditional suction (-15 to -20 cm H_2O suction)	Water seal
Brunelli 2010	RCT	Italy	Lung cancer: 100%	Digital drainage system: DigiventTM®, Millicore AB, Danderyd, Sweden	Traditional suction: Pleur Evac A®-6002-08, Teleflex Inc., Research Triangle Park, NC, USA)<br>Alternate suction (-15 cm H_2O during night and no suction during the day)

Filosso 2010	RCT	Italy	Lung cancer: 100%	Digital drainage system: Drentech Simple PLUS® P.A.L.M (REDAX,Mirandola, Italy)	Traditional wall suction(-20 cm H_2O)
Bertolaccini 2011	RCT	Italy	NR	Digital drainage system: Drentech Palm® (Redax S.r.l., Mirandola (MO),Italy	Water seal
Marjański 2013	RCT	Poland	Lung cancer: 100%	Digital drainage system: Thopaz®, Medela, Switzerland	Traditional central wall suction(-15 cm H_2O)
Brunellia 2013	RCT	Italy	Lung cancer: 100%	Digital drainage system: Thopaz®, Medela, Switzerland) (regulated individualized suction mode; -11 to -20 cm H_2O)	Water seal (regulated seal mode)(−2 cm H_2O) Thopaz® (Medela, Switzerland)
Leo 2013	RCT	Italy	NR	Digital drainage system: Thopaz®, Medela, Switzerland(-15 cm H_2O)	Water seal: Drentech®; Redax, Mirandola, Italy
Pompili 2014	RCT	Multicenter trial (Italy, the US, the UK, and China)	NR	Digital drainage system: Thopaz®, Medela, Switzerland	Traditional suction (-20 cm H_2O suction until the morning of POD 1 and water seal thereafter)
Gilbert 2015	RCT	Canada	Malignant pulmonary tumor: 92.4% Benign pulmonary tumor: 7.6%	Digital drainage system: Thopaz®, Medela, Switzerland	Water seal: Pleur Evac A®-6002-08 (Teleflex Inc., Research Triangle Park, NC, US)
Lijkendijk 2015	RCT	Denmark	NR	Digital drainage system: Thopaz® (Medela, Switzerland) (-15 cm H_2O)	Water-seal: Thora-Seal® (Covidien, Mansfield, MA, USA)
Gocyk 2016	RCT	Poland	Lung cancer: 53.94% Metastatic lung tumor: 9.06% Benign lung tumor: 14.57% Other: 22.44%	Traditional suction: Thora-Seal III® (TYCO Healthcare Ltd. Gosport, UK) (continuous -20 cmH$_2$O suction)	Water seal: Thora-Seal III® (TYCO Healthcare Ltd. Gosport, UK) (-20 cm H_2O suction until the morning of POD 1 and water seal thereafter)

Chiappetta 2017	RCT	Italy	Malignant or benign pulmonary tumor	Digital drainage system: Drentech Simple Plus PALM, Redax; Mirandola, Modena, Italy;	Water-seal
Plourde 2018	RCT	Canada	Lung tumor	Digital drainage system:ATMOS® (MedizinTechnik, Sulz, Germany)	Traditional suction: Atrium® (Oasis, AtriumMedical Corp, Hudson, NH, US) (- 20 cm H_2O)
Takamochi 2018	RCT	Japan	Lung cancer: 87.29% Metastatic lung tumor: 7.02% Benign lung tumor: 3.68% Other: 2.34%	Digital drainage system: Thopaz® (Medela Healthcare, Baar, Switzerland)	Traditional suction: Mera Sucuum MS-008EX® chest drainage system (Senko Medical, Tokyo, Japan), - 10 cm H_20

NR, not recorded; **POD**, postoperative day; **RCT**, randomized control trial; **Scenario**: the indications of pulmonary resection; **UK**, United Kingdom; **US**, United States.

Supplementary Table S4. Results of head-to-head comparison of network meta-analysis

Length of hospital stay: Head-to-head comparison

Outcome: Length of hospital stay (Measure: mean difference)		
Digital	-0.57<br>(-1.55 to 0.41)	-1.19<br>(-2.14 to -0.25)
-0.35<br>(-1.17 to 0.47)	Non-suction	-1.33<br>(-2.45 to -0.21)
-1.40<br>(-2.20 to -0.60)	-1.05<br>(-1.91 to -0.18)	Suction
Network Meta-analysis Estimate	Comparator	Direct Estimate

Teble S4.1: Outcomes for hospital stay in network meta-analysis: head-to-head comparisons
Data are presented as the mean difference with 95% CI in the column-defining treatment compared with the row-defining treatment. Comparisons should be read from left to right.
Digital, Digital chest tube drainage system; **Suction**, Suction chest tube drainage system; **Non-suction**, Non-suction chest tube drainage system; **CI**, Confident interval

Chest tube placement duration: Head-to-head comparison

Outcome: Tube placement duration (Measure: mean difference)		
Digital	-0.39<br>(-1.20 to 0.41)	-0.52<br>(-1.30 to 0.25)
-0.22<br>(-0.88 to 0.43)	Non-suction	-0.62<br>(-1.43 to 0.18)
-0.68<br>(-1.32 to -0.04)	-0.45<br>(-1.11 to 0.20)	Suction
Network Meta-analysis Estimate	Comparator	Direct Estimate

Table S4.2: Outcomes for tube replacement duration in network meta-analysis: head-to-head comparisons
Data are presented as the mean difference with 95% CI in the column-defining treatment compared with the row-defining treatment. Comparisons should be read from left to right.
Digital, Digital chest tube drainage system; **Suction**, Suction chest tube drainage system; **Non-suction**, Non-suction chest tube drainage system; **CI**, Confident interval

Prolonged air leak: Head-to-head comparison

Outcome: Prolonged air leak (Measure: odds ratio)		
Digital	0.75<br>(0.36 to 1.58)	0.82<br>(0.36 to 1.84)
0.76<br>(0.42 to 1.39)	Suction	1.04<br>(0.57 to 1.91)
0.80<br>(0.43 to 1.49)	1.05<br>(0.62 to 1.79)	Non-suction
Network Meta-analysis Estimate	Comparator	Direct Estimate

Table S4.3: Outcomes for prolonged air leak in network meta-analysis: head-to-head comparisons

Data are presented as the odds ratio with 95% CI in the column-defining treatment compared with the row-defining treatment. Comparisons should be read from left to right.

Digital, Digital chest tube drainage system; **Suction**, Suction chest tube drainage system; **Non-suction**, Non-suction chest tube drainage system; **CI**, Confident interval

Supplementary Table S5. Estimation of inconsistency

p-value: The p-value is set as 0.05. If the p-value is more than 0.05, the null hypothesis cannot be rejected and the consistency assumption could be accepted. Thus, if the p-value is less than 0.05, which means statistically significant that inconsistency exists.

Table S5.1 Estimation of inconsistency in outcome for length of hospital stay

Design-by-treatment interaction model

Q statistics to assess homogeneity / consistency

	Q	df	p-value
Total	102.65	13	< 0.0001
Within designs	98.73	12	< 0.0001
Between designs	3.92	1	0.0477

Design-specific decomposition of within-designs Q statistic

Design	Q	df	p-value
Digital:Traditional	29.61	4	< 0.0001
Digital:Water Seal	4.27	4	0.3709
Traditional:Water Seal	64.86	4	< 0.0001

Between-designs Q statistic after detaching of single designs

Detached design	Q	df	p-value
Digital:Traditional	0.00	0	--
Digital:Water Seal	0.00	0	--
Traditional:Water Seal	0.00	0	--

Q statistic to assess consistency under the assumption of a full design-by-treatment interaction random effects model

	Q	df	p-value	tau.within	tau^2.within
Between designs	0.55	1	0.4574	1.0734	1.1522

Node-splitting model

Back-calculation method to split direct and indirect evidence

```
Fixed effects model:

             comparison k prop     nma  direct  indir.    Diff     z p-value
    Digital:Traditional 5 0.83 -1.3586 -1.2522 -1.8696  0.6174  1.98  0.0477
     Digital:Water-Seal 5 0.93 -0.5405 -0.5858  0.0316 -0.6174 -1.98  0.0477
 Traditional:Water-Seal 5 0.25  0.8181  1.2838  0.6664  0.6174  1.98  0.0477

Random effects model:

             comparison k prop     nma  direct  indir.    Diff     z p-value
    Digital:Traditional 5 0.71 -1.3980 -1.1942 -1.9009  0.7067  0.78  0.4326
     Digital:Water-Seal 5 0.69 -0.3493 -0.5678  0.1389 -0.7067 -0.78  0.4326
 Traditional:Water-Seal 5 0.60  1.0488  1.3331  0.6264  0.7067  0.78  0.4326

Legend:
 comparison - Treatment comparison
 k          - Number of studies providing direct evidence
 prop       - Direct evidence proportion
 nma        - Estimated treatment effect (MD) in network meta-analysis
 direct     - Estimated treatment effect (MD) derived from direct evidence
 indir.     - Estimated treatment effect (MD) derived from indirect evidence
 Diff       - Difference between direct and indirect treatment estimates
 z          - z-value of test for disagreement (direct versus indirect)
 p-value    - p-value of test for disagreement (direct versus indirect)
```

Table S5.2 Estimation of inconsistency in outcome for chest tube placement duration

Design-by-treatment interaction model

Q statistics to assess homogeneity / consistency

	Q	df	p-value
Total	101.03	14	< 0.0001
Within designs	59.49	13	< 0.0001
Between designs	41.54	1	< 0.0001

Design-specific decomposition of within-designs Q statistic

Design	Q	df	p-value
Digital:Traditional	14.75	4	0.0052
Digital:Water Seal	1.66	4	0.7987
Traditional:Water Seal	43.09	5	< 0.0001

Between-designs Q statistic after detaching of single designs

Detached design	Q	df	p-value
Digital:Traditional	0.00	0	--
Digital:Water Seal	0.00	0	--
Traditional:Water Seal	0.00	0	--

Q statistic to assess consistency under the assumption of a full design-by-treatment interaction random effects model

	Q	df	p-value	tau.within	tau^2.within
Between designs	0.98	1	0.3202	0.6436	0.4142

Node-splitting model

Back-calculation method to split direct and indirect evidence

Fixed effects model:

comparison	k	prop	nma	direct	indir.	Diff	z	p-value
Digital:Traditional	5	0.78	-1.0396	-0.7291	-2.1186	1.3895	6.45	< 0.0001
Digital:Water-Seal	5	0.69	-0.0595	-0.4959	0.8936	-1.3895	-6.45	< 0.0001
Traditional:Water-Seal	6	0.54	0.9801	1.6227	0.2332	1.3895	6.45	< 0.0001

Random effects model:

comparison	k	prop	nma	direct	indir.	Diff	z	p-value
Digital:Traditional	5	0.68	-0.6797	-0.5237	-1.0167	0.4930	0.70	0.4824
Digital:Water-Seal	5	0.66	-0.2248	-0.3938	0.0992	-0.4930	-0.70	0.4824
Traditional:Water-Seal	6	0.66	0.4549	0.6229	0.1299	0.4930	0.70	0.4824

Legend:
comparison - Treatment comparison
k - Number of studies providing direct evidence
prop - Direct evidence proportion
nma - Estimated treatment effect (MD) in network meta-analysis
direct - Estimated treatment effect (MD) derived from direct evidence
indir. - Estimated treatment effect (MD) derived from indirect evidence
Diff - Difference between direct and indirect treatment estimates
z - z-value of test for disagreement (direct versus indirect)
p-value - p-value of test for disagreement (direct versus indirect)

Table S5.3 Estimation of inconsistency in outcome for prolonged air leak

Design-by-treatment interaction model

Q statistics to assess homogeneity / consistency

	Q	df	p-value
Total	20.31	12	0.0614
Within designs	20.16	11	0.0432
Between designs	0.17	1	0.6946

Design-specific decomposition of within-designs Q statistic

Design	Q	df	p-value
Digital:Traditional	6.78	3	0.0791
Digital:Water Seal	0.62	2	0.7343
Traditional:Water Seal	12.76	6	0.0470

Between-designs Q statistic after detaching of single designs

Detached design	Q	df	p-value
Digital:Traditional	0.00	0	--
Digital:Water Seal	0.00	0	--
Traditional:Water Seal	0.00	0	--

Q statistic to assess consistency under the assumption of a full design-by-treatment interaction random effects model

	Q	df	p-value	tau.within	tau^2.within
Between designs	0.00	1	0.9788	0.5526	0.3053

Node-splitting model

Back-calculation method to split direct and indirect evidence

```
Fixed effects model:

            comparison k prop    nma direct indir.    RoR     z p-value
   Digital:Traditional 4 0.63 0.6885 0.6475 0.7643 0.8473 -0.39  0.6946
    Digital:Water-Seal 3 0.66 0.7204 0.7626 0.6461 1.1802  0.39  0.6946
Traditional:Water-Seal 7 0.71 1.0464 0.9978 1.1776 0.8473 -0.39  0.6946

Random effects model:

            comparison k prop    nma direct indir.    RoR     z p-value
   Digital:Traditional 4 0.65 0.7634 0.7496 0.7891 0.9499 -0.08  0.9362
    Digital:Water-Seal 3 0.59 0.8017 0.8189 0.7779 1.0527  0.08  0.9362
Traditional:Water-Seal 7 0.77 1.0502 1.0377 1.0924 0.9499 -0.08  0.9362

Legend:
 comparison - Treatment comparison
 k          - Number of studies providing direct evidence
 prop       - Direct evidence proportion
 nma        - Estimated treatment effect (OR) in network meta-analysis
 direct     - Estimated treatment effect (OR) derived from direct evidence
 indir.     - Estimated treatment effect (OR) derived from indirect evidence
 RoR        - Ratio of Ratios (direct versus indirect)
 z          - z-value of test for disagreement (direct versus indirect)
 p-value    - p-value of test for disagreement (direct versus indirect)
```

Supplementary Table S6. Risk of bias table of included studies

Table. Risk of bias in included studies

First author, year	Random Sequence generation (Selection bias)	Allocation Concealment (Selection bias)	Blinding of participants and personnel (Performance bias)	Blinding of outcome assessment (Detection bias)	Incomplete outcome data (Attrition bias)	Selective reporting (Reporting bias)	Other bias
Marshall, 2002	High	High	High	High	Low	Unclear	Low
Ayed, 2003	Unclear	Unclear	High	High	Low	Low	Low
Brunelli, 2004	Unclear	Unclear	High	High	Low	Low	Low
Brunelli, 2005	Low	High	High	High	Low	Low	Low
Alphonso,2005	Low	Unclear	High	High	Unclear	Unclear	Low
Kakhki, 2006	High	Unclear	High	High	Low	Low	Unclear
Cerfolio, 2008	Low	Unclear	High	High	Low	Low	Low
Prokakis, 2008	Low	Unclear	High	High	Low	Low	Low
Brunelli, 2010	Unclear	Unclear	High	High	Low	Low	Low
Filosso, 2010	Unclear	High	High	High	Low	Low	Low
Bertolaccini, 2011	Low	Unclear	High	High	Low	Low	Low
Marjański, 2013	Low	Unclear	High	High	Low	Low	Low
Brunelli, 2013	Low	Unclear	High	High	Low	Low	Low
Leo, 2013	High	Unclear	High	High	Low	Low	Low
Pompili, 2014	Low	Unclear	High	Unclear	Low	Low	Low
Gilbert, 2015	Low	Unclear	High	High	Low	Low	Low
Lijkendijk, 2015	Low	Unclear	High	High	Low	Low	Low
Gocyk, 2016	Low	Unclear	High	High	Low	Low	Low
Chiappetta, 2017	Unclear	Unclear	High	High	Low	Low	Low
Plourde, 2018	Low	High	High	High	Low	Low	High
Takamochi, 2018	Unclear	Unclear	High	High	Low	Low	Low

Supplementary Table S7. Sensitivity analyses

p-value: The p-value is set as 0.05. If the p-value is more than 0.05, the null hypothesis cannot be rejected and the consistency assumption could be accepted. Thus, if the p-value is less than 0.05, which means statistically significant that inconsistency exists.

Table S7.1 Sensitivity analyses regarding the estimation of inconsistency in outcome for length of hospital stay

Design-by-treatment interaction model

Q statistics to assess homogeneity / consistency

	Q	df	p-value
Total	10.51	9	0.3111
Within designs	7.08	8	0.5282
Between designs	3.43	1	0.0641

Design-specific decomposition of within-designs Q statistic

Design	Q	df	p-value
Digital:Traditional	4.27	4	0.3708
Digital:Water Seal	2.14	2	0.3432
Traditional:Water Seal	0.67	2	0.7152

Between-designs Q statistic after detaching of single designs

Detached design	Q	df	p-value
Digital:Traditional	0.00	0	--
Digital:Water Seal	0.00	0	--
Traditional:Water Seal	0.00	0	--

Q statistic to assess consistency under the assumption of a full design-by-treatment interaction random effects model

	Q	df	p-value	tau.within	tau^2.within
Between designs	3.43	1	0.0641	0	0

Node-splitting model

Back-calculation method to split direct and indirect evidence

```
Fixed effects model:

          comparison k prop     nma  direct  indir.    Diff     z p-value
 Digital:Non-suction 5 0.94 -0.5484 -0.5858  0.0686 -0.6543 -1.85  0.0641
     Digital:Suction 3 0.82 -1.0660 -0.9465 -1.6008  0.6543  1.85  0.0641
 Non-suction:Suction 3 0.24 -0.5176 -1.0151 -0.3607 -0.6543 -1.85  0.0641

Random effects model:

          comparison k prop     nma  direct  indir.    Diff     z p-value
 Digital:Non-suction 5 0.88 -0.4862 -0.5660  0.0855 -0.6515 -1.58  0.1132
     Digital:Suction 3 0.78 -1.0612 -0.9169 -1.5684  0.6515  1.58  0.1132
 Non-suction:Suction 3 0.34 -0.5750 -1.0024 -0.3509 -0.6515 -1.58  0.1132

Legend:
 comparison - Treatment comparison
 k          - Number of studies providing direct evidence
 prop       - Direct evidence proportion
 nma        - Estimated treatment effect (MD) in network meta-analysis
 direct     - Estimated treatment effect (MD) derived from direct evidence
 indir.     - Estimated treatment effect (MD) derived from indirect evidence
 Diff       - Difference between direct and indirect treatment estimates
 z          - z-value of test for disagreement (direct versus indirect)
 p-value    - p-value of test for disagreement (direct versus indirect)
```

Table S7.2 Sensitivity analyses regarding the estimation of inconsistency in outcome for chest tube placement duration

Design-by-treatment interaction model

Q statistics to assess homogeneity / consistency

	Q	df	p-value
Total	7.81	10	0.6478
Within designs	6.55	9	0.6840
Between designs	1.26	1	0.2622

Design-specific decomposition of within-designs Q statistic

Design	Q	df	p-value
Digital:Traditional	1.66	4	0.7987
Digital:Water Seal	0.74	2	0.6904
Traditional:Water Seal	4.15	3	0.2456

Between-designs Q statistic after detaching of single designs

Detached design	Q	df	p-value
Digital:Traditional	0.00	0	--
Digital:Water Seal	0.00	0	--
Traditional:Water Seal	0.00	0	--

Q statistic to assess consistency under the assumption of a full design-by-treatment interaction random effects model

	Q	df	p-value	tau.within	tau^2.within
Between designs	1.26	1	0.2622	0	0

Node-splitting model

Back-calculation method to split direct and indirect evidence

```
Fixed effects model:

          comparison k prop     nma  direct  indir.    Diff     z p-value
 Digital:Non-suction 5 0.86 -0.4448 -0.4959 -0.1369 -0.3590 -1.12  0.2622
     Digital:Suction 3 0.85 -1.0324 -0.9790 -1.3381  0.3590  1.12  0.2622
 Non-suction:Suction 4 0.29 -0.5876 -0.8422 -0.4832 -0.3590 -1.12  0.2622

Random effects model:

          comparison k prop     nma  direct  indir.    Diff     z p-value
 Digital:Non-suction 5 0.86 -0.4448 -0.4959 -0.1369 -0.3590 -1.12  0.2622
     Digital:Suction 3 0.85 -1.0324 -0.9790 -1.3381  0.3590  1.12  0.2622
 Non-suction:Suction 4 0.29 -0.5876 -0.8422 -0.4832 -0.3590 -1.12  0.2622

Legend:
 comparison - Treatment comparison
 k          - Number of studies providing direct evidence
 prop       - Direct evidence proportion
 nma        - Estimated treatment effect (MD) in network meta-analysis
 direct     - Estimated treatment effect (MD) derived from direct evidence
 indir.     - Estimated treatment effect (MD) derived from indirect evidence
 Diff       - Difference between direct and indirect treatment estimates
 z          - z-value of test for disagreement (direct versus indirect)
 p-value    - p-value of test for disagreement (direct versus indirect)
```

<u>Table S7.3 Sensitivity analyses regarding the estimation of inconsistency in outcome for prolonged air leak</u>

Design-by-treatment interaction model

Q statistics to assess homogeneity / consistency

	Q	df	p-value
Total	15.57	11	0.1577
Within designs	15.57	10	0.1126
Between designs	0.00	1	0.9529

Design-specific decomposition of within-designs Q statistic

Design	Q	df	p-value
Digital:Traditional	0.62	2	0.7343
Digital:Water Seal	6.78	3	0.0791
Traditional:Water Seal	8.17	5	0.1471

Between-designs Q statistic after detaching of single designs

Detached design	Q	df	p-value
Digital:Traditional	0.00	0	--
Digital:Water Seal	0.00	0	--
Traditional:Water Seal	0.00	0	--

Q statistic to assess consistency under the assumption of a full design-by-treatment interaction random effects model

	Q	df	p-value	tau.within	tau^2.within
Between designs	0.05	1	0.8239	0.4444	0.1974

Node-splitting model

Back-calculation method to split direct and indirect evidence

```
Fixed effects model:

          comparison k prop    nma direct indir.    RoR     z p-value
 Digital:Non-suction 3 0.67 0.7562 0.7626 0.7436 1.0255  0.06  0.9529
     Digital:Suction 4 0.64 0.6535 0.6475 0.6641 0.9751 -0.06  0.9529
 Non-suction:Suction 6 0.70 0.8642 0.8708 0.8492 1.0255  0.06  0.9529

Random effects model:

          comparison k prop    nma direct indir.    RoR     z p-value
 Digital:Non-suction 3 0.61 0.8332 0.8036 0.8813 0.9118 -0.16  0.8724
     Digital:Suction 4 0.66 0.6938 0.7162 0.6531 1.0967  0.16  0.8724
 Non-suction:Suction 6 0.74 0.8327 0.8127 0.8912 0.9118 -0.16  0.8724

Legend:
 comparison - Treatment comparison
 k          - Number of studies providing direct evidence
 prop       - Direct evidence proportion
 nma        - Estimated treatment effect (OR) in network meta-analysis
 direct     - Estimated treatment effect (OR) derived from direct evidence
 indir.     - Estimated treatment effect (OR) derived from indirect evidence
 RoR        - Ratio of Ratios (direct versus indirect)
 z          - z-value of test for disagreement (direct versus indirect)
 p-value    - p-value of test for disagreement (direct versus indirect)
```

Supplementary Table S8. Reference list of included studies.

Article	Reason
Graham,1992[1]	No operation(Trauma, thoracotomy)
Waldhausen, 2002[2]	No lung resection in all patients(children, multiple kind of operation , suction and waterseal(prospective, randomize))
Vuorisalo 2005[3]	Only Comparing device(suction and valve drainage bag(prospective, randomize))
Varela 2008[4]	Limited data without available result(indication to remove chest tubes, no detail data(prospective, randomize))
Lijkendijk, 2014[5]	Limited data without available result(Only abstract)
Waele, 2014[6]	Limited data without available result(Only abstract)
Mier,2016[7]	No operation
Barozzi, 2018[8]	Limited data without available result(Thopaz + self-contained drainage system vs wall suction)
Cui 2019[9]	Only Comparing device(chest tube and suction ball (prospective, randomize))
Marulli, 2019[10]	Limited data without available result(No available result)
Ruigrok, 2020[11]	No lung resection in all patients(spontaneous pneumothorax, no lung resection (prospective, randomize))

1. Graham ANJ, Cosgrove AP, Gibbons JRP, McGuigan JA. Randomised clinical trial of chest drainage systems. *Thorax.* 1992;47:461-462.

2. Waldhausen JHT, Cusick RA, Graham DD, Pittinger TP, Sawin RS. Removal of chest tubes in children without water seal after elective thoracic procedures: A randomized prospective study. *Journal of the American College of Surgeons.* 2002;194:411-415.

3. Vuorisalo S, Aarnio P, Hannukainen J. Comparison between flutter valve drainage bag and underwater seal device for pleural drainage after lung surgery. *Scandinavian Journal of Surgery.* 2005;94:56-58.

4. Varela G, Jiménez MF, Novoa NM, Aranda JL. Postoperative chest tube management: measuring air leak using an electronic device decreases variability in the clinical practice. *Eur J Cardiothorac Surg.* 2009;35:28-31.

5. Lijkendijk M, Licht PB, Neckelmann K. Digital versus analogue chest tube drainage following lobectomy: A randomised trial. *Interactive Cardiovascular and Thoracic Surgery.* 2014;19:S13.

6. De Waele M, Agzarian J, Hanna WC, et al. Does the usage of digital chest drainage systems reduce pleural inflammation and volume of pleural effusion following oncologic pulmonary resection?-A prospective randomized trial. *J Thorac Dis.* 2017;9:1598-1606.

7. Mier JM, Cortés-Julián G, Berrios-Mejía J, Víctor-Valdivia Z. [The benefits of digital chest drainage in pleural decortication in thoracic empyema. Prospective, randomized, control trial]. *Cir Cir.* 2017;85:522-525.

8. Barozzi L, Biagio LS, Meneguzzi M, et al. Do We Still Need Wall Suction for Chest Drainage? *Heart, Lung and Circulation.* 2018;27:S502.

9. Cui Z, Zhang Y, Xu C, et al. Comparison of the results of two chest tube managements during an enhanced recovery program after video-assisted thoracoscopic lobectomy: A randomized trial. *Thorac Cancer.* 2019;10:1993-1999.

10. Marulli G, Comacchio GM, Nosotti M, et al. Multicenter randomized study on the comparison between electronic and traditional chest drainage systems. *Trials.* 2019;20:730.
11. Ruigrok D, Kunst PWA, Blacha MMJ, et al. Digital versus analogue chest drainage system in patients with primary spontaneous pneumothorax: a randomized controlled trial. *BMC Pulm Med.* 2020;20:136.

Supplementary Table S9. Reference list of included studies.

Reference list of included studies
1. Marshall MB, Deeb ME, Bleier JI, et al. Suction vs water seal afterpulmonary resection: a randomized prospective study. Chest 2002;121(3):831-5 doi:10.1378/chest.121.3.831
2. Ayed AK. Suction versus water seal after thoracoscopy for primary spontaneous pneumothorax: prospective randomized study. Ann Thorac Surg2003;75(5):1593-6 doi: 10.1016/s0003-4975(02)04894-4
3. Brunelli A, Monteverde M, Borri A, et al. Comparison of water seal and suction after pulmonary lobectomy: a prospective, randomized trial. Ann Thorac Surg2004;77(6):1932-7; discussion 37 doi:10.1016/j.athoracsur.2003.12.022
4. Alphonso N, Tan C, Utley M, et al. A prospective randomized controlled trial of suction versus non-suction to the under-water seal drains following lung resection. Eur J Cardiothorac Surg 2005;27(3):391-4 doi:10.1016/j.ejcts.2004.12.004
5. Brunelli A, Sabbatini A, Xiume F, Refai MA, Salati M, Marasco R. Alternate suction reduces prolonged air leak after pulmonary lobectomy: a randomized comparison versus water seal. Ann Thorac Surg 2005;80(3):1052-5 doi:10.1016/j.athoracsur.2005.03.073
6. Daneshvar Kakhki A, Pooya M, Pejhan S, et al. Effect of chest tube suction on air-leak following lung resection. Tanaffos 2006;5(1):37-43
7. Cerfolio RJ, Bryant AS. The benefits of continuous and digital air leak assessment after elective pulmonary resection: a prospective study. Ann Thorac Surg 2008;86(2):396-401 doi:10.1016/j.athoracsur.2008.04.016
8. Prokakis C, Koletsis EN, Apostolakis E, et al. Routine suction of intercostal drains is not necessary after lobectomy: a prospective randomized trial. World J Surg 2008;32(11):2336-42 doi: 10.1007/s00268-008-9741-3
9. Brunelli A, Salati M, Refai M, Di Nunzio L, Xiume F, Sabbatini A. Evaluation of a new chest tube removal protocol using digital air leak monitoring after lobectomy: a prospective randomised trial. Eur J Cardiothorac Surg 2010;37(1):56-60 doi: 10.1016/j.ejcts.2009.05.006
10. Filosso PL, Ruffini E, Solidoro P, Molinatti M, Bruna MC, Oliaro A. Digital air leak monitoring after lobectomy for primary lung cancer in patients with moderate COPD: can a fast-tracking algorithm reduce postoperative costs and complications? J Cardiovasc Surg (Torino) 2010;51(3):429-33
11. Bertolaccini L, Rizzardi G, Filice MJ, Terzi A. Six sigma approach - an objective strategy in digital assessment of postoperative air leaks: a prospective randomised study. Eur J Cardiothorac Surg 2011;39(5):e128-32 doi:10.1016/j.ejcts.2010.12.027
12. Brunelli A, Salati M, Pompili C, Refai M, Sabbatini A. Regulated tailored suction vs regulated seal: a prospective randomized trial on air leak duration. Eur J Cardiothorac Surg 2013;43(5):899-904 doi: 10.1093/ejcts/ezs518
13. Leo F, Duranti L, Girelli L, et al. Does external pleural suction reduce prolonged air leak after lung resection? Results from the AirINTrial after 500 randomized cases. Ann Thorac Surg 2013;96(4):1234-39 doi:10.1016/j.athoracsur.2013.04.079
14. Marjański T, Sternau A, Rzyman W. THORACIC SURGERYThe implementation of a digital chest drainage system significantly reduces complication rates after lobectomy – a randomized clinical trial. Kardiochirurgia i Torakochirurgia Polska/Polish Journal of Thoracic and Cardiovascular Surgery 2013;10(2):133-38 doi: 10.5114/kitp.2013.36133
15. Pompili C, Detterbeck F, Papagiannopoulos K, et al. Multicenter international randomized comparison of objective and subjective outcomes between electronic and traditional chest drainage systems. Ann Thorac Surg 2014;98(2):490-6; discussion 96-7 doi:10.1016/j.athoracsur.2014.03.043
16. Lijkendijk M, Licht PB, Neckelmann K. Electronic versus traditional chest tube drainage following lobectomy: a randomized trial. Eur J Cardiothorac Surg 2015;48(6):893-8; discussion 98 doi:10.1093/ejcts/ezu535
17. Gilbert S, McGuire AL, Maghera S, et al. Randomized trial of digital versus analog pleural drainage in patients with or without a pulmonary air leak after lung resection. J Thorac Cardiovasc Surg 2015;150(5):1243-9 doi:10.1016/j.jtcvs.2015.08.051
18. Gocyk W, Kużdżał J, Włodarczyk J, et al. Comparison of Suction Versus Nonsuction Drainage After Lung Resections: A Prospective Randomized Trial. Ann Thorac Surg 2016;102(4):1119-24 doi: 10.1016/j.athoracsur.2016.04.066
19. Chiappetta M, Lococo F, Nachira D, et al. Digital Devices Improve Chest Tube Management: Results from a Prospective Randomized Trial. Thorac Cardiovasc Surg 2018;66(7):595-602 doi: 10.1055/s-0037-1607443
20. Plourde M, Jad A, Dorn P, et al. Digital Air Leak Monitoring for Lung Resection Patients: A Randomized Controlled Clinical Trial. Ann Thorac Surg 2018;106(6):1628-32 doi:10.1016/j.athoracsur.2018.06.080
21. Takamochi K, Nojiri S, Oh S, et al. Comparison of digital and traditional thoracic drainage systems for postoperative chest tube management after pulmonary resection: A prospective randomized trial. J Thorac Cardiovasc Surg 2018;155(4):1834-40 doi: 10.1016/j.jtcvs.2017.09.145

Supplementary Figure

Promising Effects of Digital Chest Tube Drainage System for Pulmonary Resection: A Systematic Review and Network Meta-Analysis

Po-Chih Chang[1,2,3,4], Kai-Hua Chen[5#], Hong-Jie Jhou[6], Cho-Hao Lee[7], Shah-Hwa Chou[1,8], Po-Huang Chen[9+], Ting-Wei Chang[5*]

Contents

Supplementary Figure S1. Comparison direct and indirect evidence in network meta-analysis

Supplementary Figure S2. GRADE approach for rating the quality of treatment effect estimate

Supplementary Figure S3. Results of network meta-analysis and net-split comparison

Supplementary Figure S4. Comparison-adjusted funnel plots and Egger's test

Supplementary Figure S5. Sensitivity analyses for the outcomes in network meta-analysis

Supplementary Figure S1. Comparison direct and indirect evidence in network meta-analysis

<u>Length of hospital stay:</u>

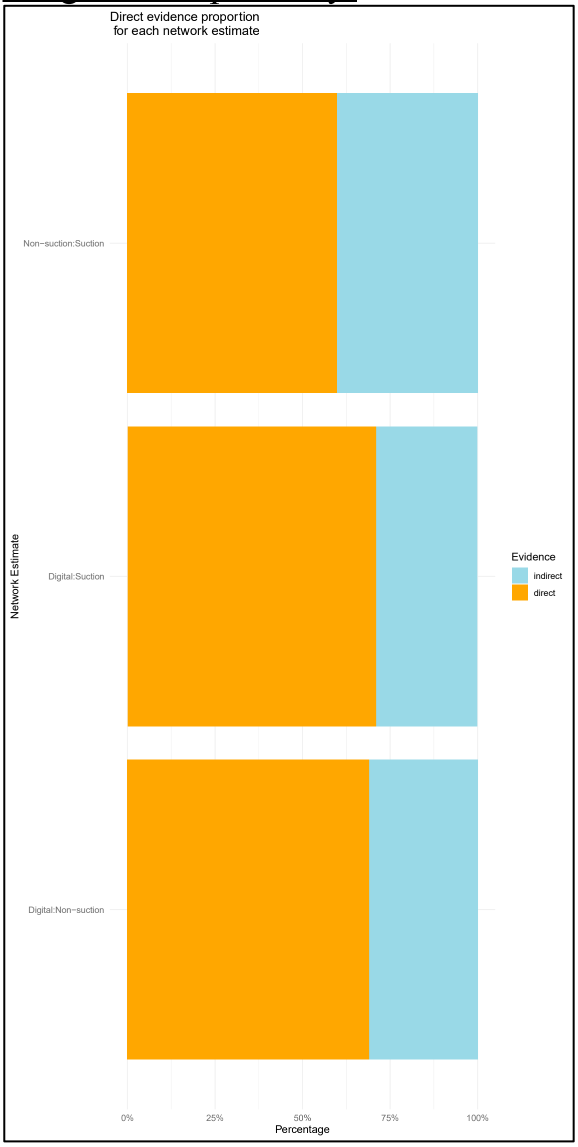

Supplementary Figure S1.1 Comparison direct and indirect evidence in network meta-analysis: Length of hospital stay

Digital, Digital chest tube drainage system; **Suction**, Suction chest tube drainage system; Non-suction, Non-suction chest tube drainage system.

<u>Chest tube placement duration:</u>

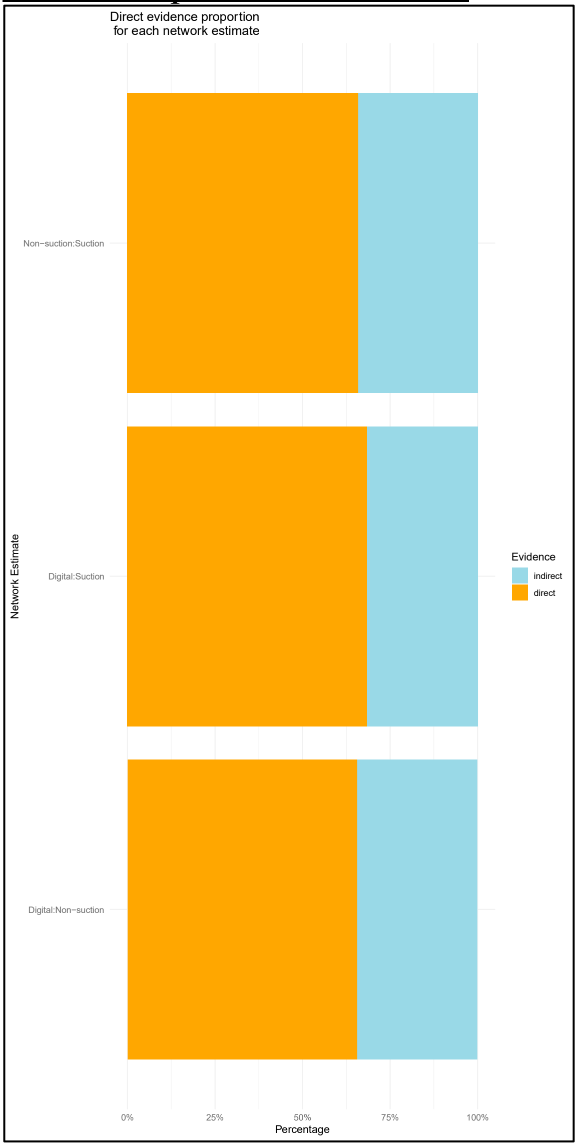

Supplementary Figure S1.2 Comparison direct and indirect evidence in network meta-analysis: Chest tube placement duration.
Digital, Digital chest tube drainage system; **Suction**, Suction chest tube drainage system; Non-suction, Non-suction chest tube drainage system.

<u>Prolonged air leak:</u>

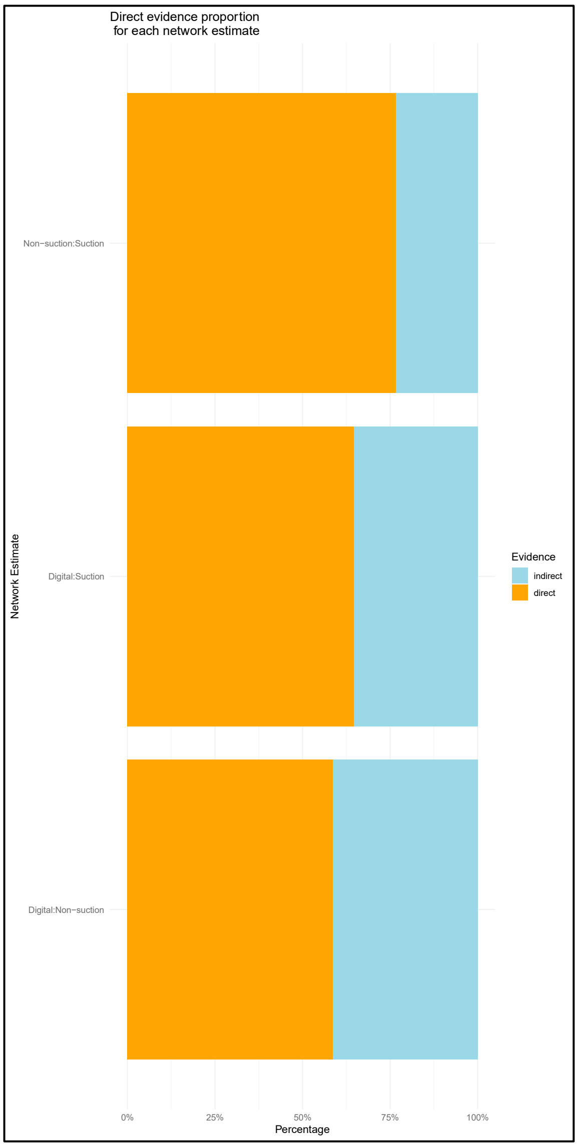

Supplementary Figure S1.3 Comparison direct and indirect evidence in network meta-analysis: Prolonged air leak.

Digital, Digital chest tube drainage system; **Suction**, Suction chest tube drainage system; Non-suction, Non-suction chest tube drainage system.

Supplementary Figure S2. GRADE approach for rating the quality of treatment effect estimate

Supplementary Figure S2.1 Length of hospital stay

Summary of Evidence:

Promising Effects of Digital Chest Tube Drainage System for Pulmonary Resection: A Systematic Review and Network Meta-Analysis

Primary Outcome: Hospital stay

	Direct evidence		Indirect evidence		Network meta-analysis	
Comparison	Mean difference (95% CI)	Quality of evidence	Mean difference (95% CI)	Quality of evidence	Mean difference (95% CI)	Quality of evidence
Digital *v* Suction	-1.19 (-2.14 to -0.24)	**Low***	-1.90 (-3.39 to -0.41)	**Low**	-1.40 (-2.20 to -0.60)	**Low**
Digital *v* Non-suction	-0.57 (-1.55 to 0.41)	**High**	0.14 (-1.33 to 1.61)	**Low**	-0.35 (-1.17 to 0.47)	**High**
Suction *v* Non-suction	1.33 (0.21 to 2.45)	**Low***	0.63 (-0.74 to 1.99)	**Low**	1.05 (0.18 to 1.91)	**Low**

CI: Confidence interval; **Digital**, Digital chest drainage system; **Traditional**, Traditional chest drainage system; **Water-seal**, Water-seal chest drainage system

GRADE Working Group grades of evidence
High quality: We are very confident that the true effect lies close to that of the estimate of the effect
Moderate quality: We are moderately confident in the effect estimate: The true effect is likely to be close to the estimate of the effect, but there is a possibility that it is substantially different
Low quality: Our confidence in the effect estimate is limited: The true effect may be substantially different from the estimate of the effect
Very low quality: We have very little confidence in the effect estimate: The true effect is likely to be substantially different from the estimate of effect

Explanations

*Limitations (risk of bias). †Inconsistency. ‡Imprecision. ¶ Indirectness because of questionable comparability of trial populations to target population of NMA or questionable outcome measurement, or intransitivity. § Greater precision. §§ Cannot be estimated because the intervention was not connected in a loop in the evidence network.

Supplementary Figure S2.2 Chest tube placement duration

Summary of Evidence:

Promising Effects of Digital Chest Tube Drainage System for Pulmonary Resection: A Systematic Review and Network Meta-Analysis

Secondary Outcome: Tube placement duration

	Direct evidence		Indirect evidence		Network meta-analysis	
Comparison	Mean difference (95% CI)	Quality of evidence	Mean difference (95% CI)	Quality of evidence	Mean difference (95% CI)	Quality of evidence
Digital *v* Suction	-0.52 (-1.30 to 0.25)	**Low***	-1.02 (-2.15 to 0.12)	**Moderate**	-0.68 (-1.32 to -0.04)	**Moderate**
Digital *v* Non-suction	-0.39 (-1.20 to 0.41)	**High**	0.10 (-1.02 to 1.21)	**High**	-0.22 (-0.88 to 0.43)	**High**
Suction *v* Non-suction	0.62 (-0.18 to 1.43)	**Moderate***	0.13 (-0.99 to 1.25)	**Low**	0.45 (-0.20 to 1.11)	**Moderate**

CI: Confidence interval; **Digital**, Digital chest drainage system; **Traditional**, Traditional chest drainage system; **Water-seal**, Water-seal chest drainage system

GRADE Working Group grades of evidence
High quality: We are very confident that the true effect lies close to that of the estimate of the effect
Moderate quality: We are moderately confident in the effect estimate: The true effect is likely to be close to the estimate of the effect, but there is a possibility that it is substantially different
Low quality: Our confidence in the effect estimate is limited: The true effect may be substantially different from the estimate of the effect
Very low quality: We have very little confidence in the effect estimate: The true effect is likely to be substantially different from the estimate of effect

Explanations

*Limitations (risk of bias). †Inconsistency. ‡Imprecision. ¶ Indirectness because of questionable comparability of trial populations to target population of NMA or questionable outcome measurement, or intransitivity. § Greater precision. §§ Cannot be estimated because the intervention was not connected in a loop in the evidence network.

Supplementary Figure S2.3 Prolonged air leak

Summary of Evidence:

Promising Effects of Digital Chest Tube Drainage System for Pulmonary Resection: A Systematic Review and Network Meta-Analysis

Secondary Outcome: Prolonged air leak

	Direct evidence		Indirect evidence		Network meta-analysis	
Comparison	Odds ratio (95% CI)	Quality of evidence	Odds ratio (95% CI)	Quality of evidence	Odds ratio (95% CI)	Quality of evidence
Digital *v* Suction	0.75 (0.36 to 1.58)	**Moderate***	0.79 (0.29 to 2.17)	**Moderate**	0.76 (0.42 to 1.39)	**Moderate**
Digital *v* Non-suction	0.82 (0.36 to 1.83)	**High**	0.78 (0.30 to 2.04)	**High**	0.80 (0.43 to 1.49)	**High**
Suction *v* Non-suction	1.04 (0.57 to 1.91)	**Low***	1.09 (0.36 to 3.29)	**Low**	1.05 (0.62 to 1.79)	**Moderate**

CI: Confidence interval; **Digital**, Digital chest drainage system; **Traditional**, Traditional chest drainage system; **Water-seal**, Water-seal chest drainage system

GRADE Working Group grades of evidence
High quality: We are very confident that the true effect lies close to that of the estimate of the effect
Moderate quality: We are moderately confident in the effect estimate: The true effect is likely to be close to the estimate of the effect, but there is a possibility that it is substantially different
Low quality: Our confidence in the effect estimate is limited: The true effect may be substantially different from the estimate of the effect
Very low quality: We have very little confidence in the effect estimate: The true effect is likely to be substantially different from the estimate of effect

Explanations

*Limitations (risk of bias). †Inconsistency. ‡Imprecision. ¶ Indirectness because of questionable comparability of trial populations to target population of NMA or questionable outcome measurement, or intransitivity. § Greater precision. §§ Cannot be estimated because the intervention was not connected in a loop in the evidence network.

Supplementary Figure S3. Results of network meta-analysis and net-split comparison

Length of hospital stay

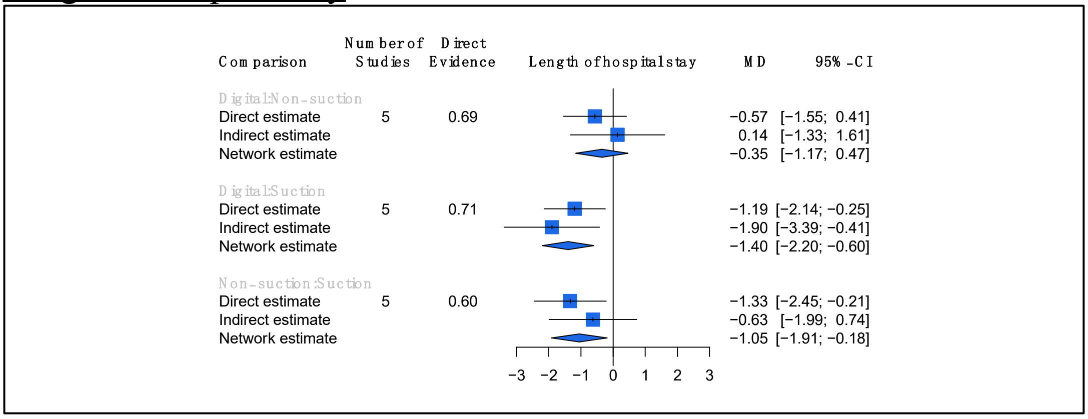

Supplementary Figure S3.1 Results of network meta-analysis and net-split comparison: Length of hospital stay

Digital, Digital chest tube drainage system; **Suction**, Suction chest tube drainage system; **Non-suction**, Non-suction chest tube drainage system; **MD**, Mean difference; **CI**, Confident interval.

Chest tube placement duration

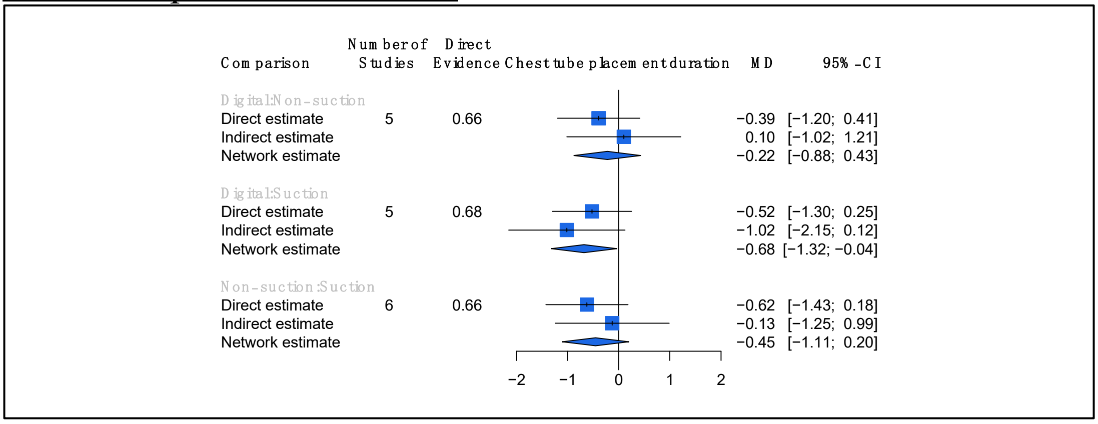

Supplementary Figure S3.2 Results of network meta-analysis and net-split comparison: Chest tube placement duration

Digital, Digital chest tube drainage system; **Suction**, Suction chest tube drainage system; **Non-suction**, Non-suction chest tube drainage system; **MD**, Mean difference; **CI**, Confident interval.

Prolonged air leak

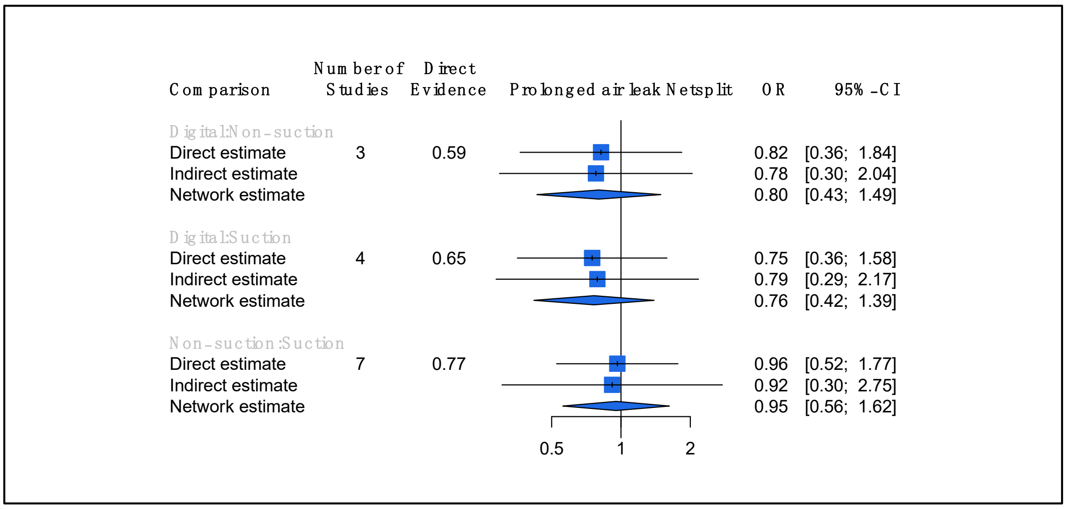

Supplementary Figure S3.3 Results of network meta-analysis and net-split comparison: Prolonged air leak

Digital, Digital chest tube drainage system; **Suction**, Suction chest tube drainage system; **Non-suction**, Non-suction chest tube drainage system; **OR**, odds ratio; **CI**, Confident interval.

Supplementary Figure S4. Comparison-adjusted funnel plots and Egger's test

<u>Length of hospital stay:</u>

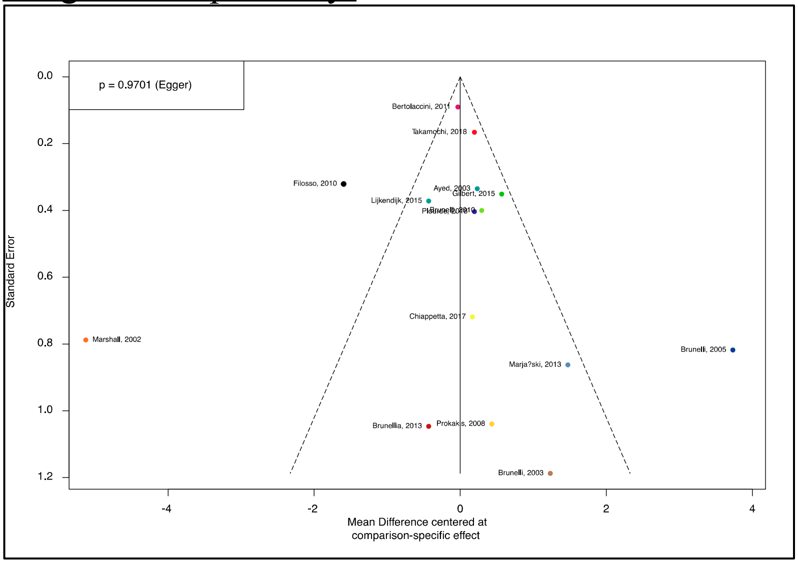

Supplementary Figure S4.1 Comparison-adjusted funnel plot in outcome for length of hospital stay:

Digital, Digital chest tube drainage system; **Suction**, Suction chest tube drainage system; Non-suction, Non-suction chest tube drainage system.

<u>Chest tube placement duration:</u>

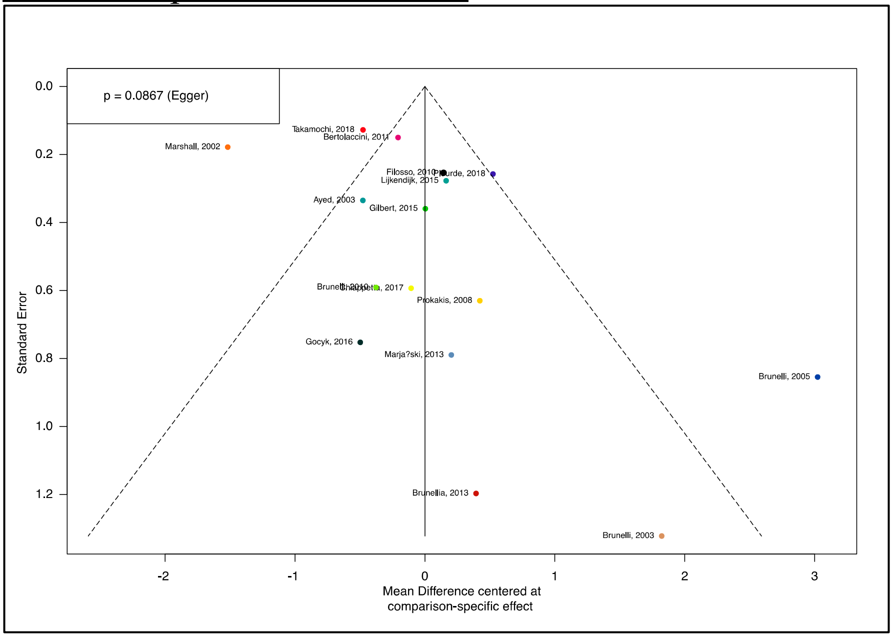

Supplementary Figure S4.2 Comparison-adjusted funnel plot in outcome for chest tube placement duration:

Digital, Digital chest tube drainage system; **Suction**, Suction chest tube drainage system; **Non-suction,** Non-suction chest tube drainage system.

Prolonged air leak:

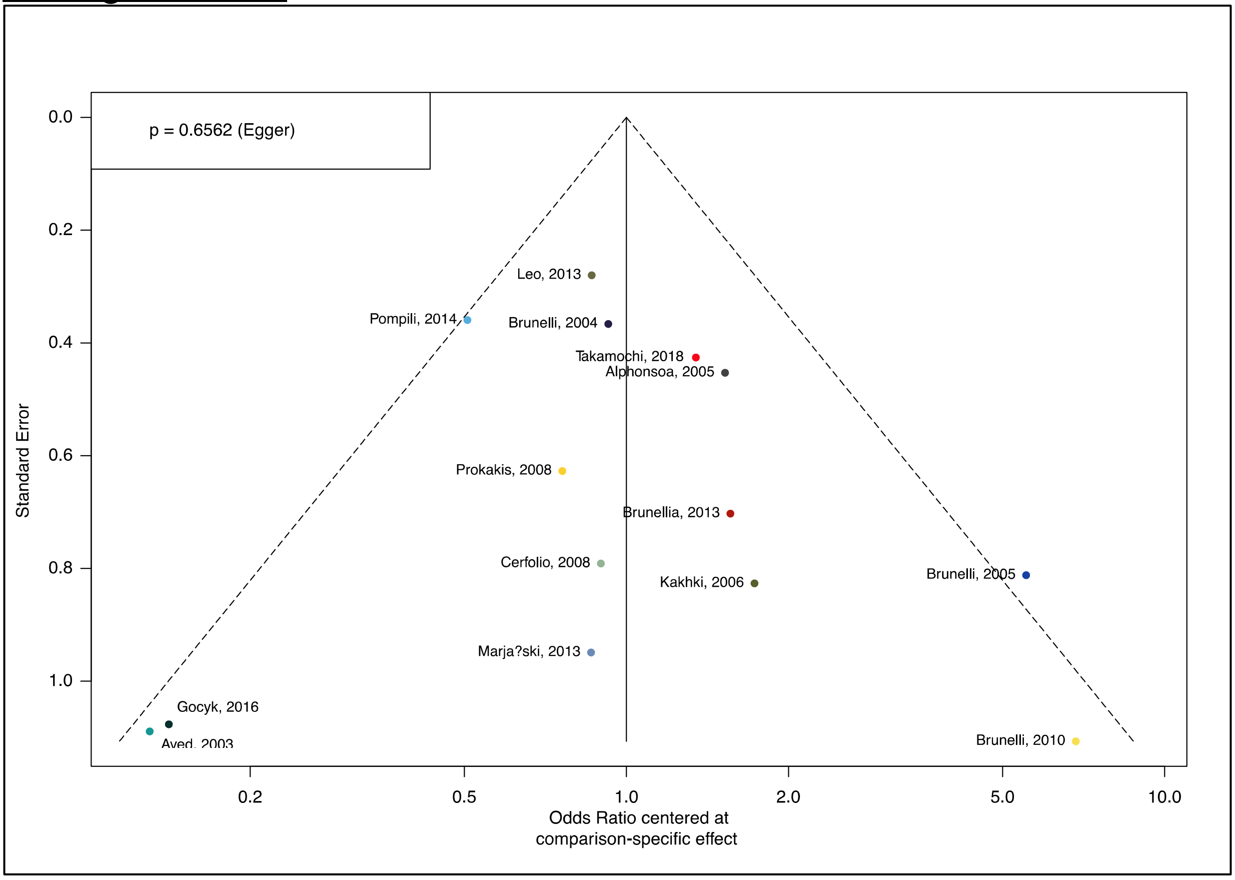

Supplementary Figure S4.3 Comparison-adjusted funnel plot in outcome for prolonged air leak:

Digital, Digital chest tube drainage system; **Suction**, Suction chest tube drainage system; **Non-suction,** Non-suction chest tube drainage system.

Supplementary Figure S5. Sensitivity analyses for the outcomes in network meta-analysis

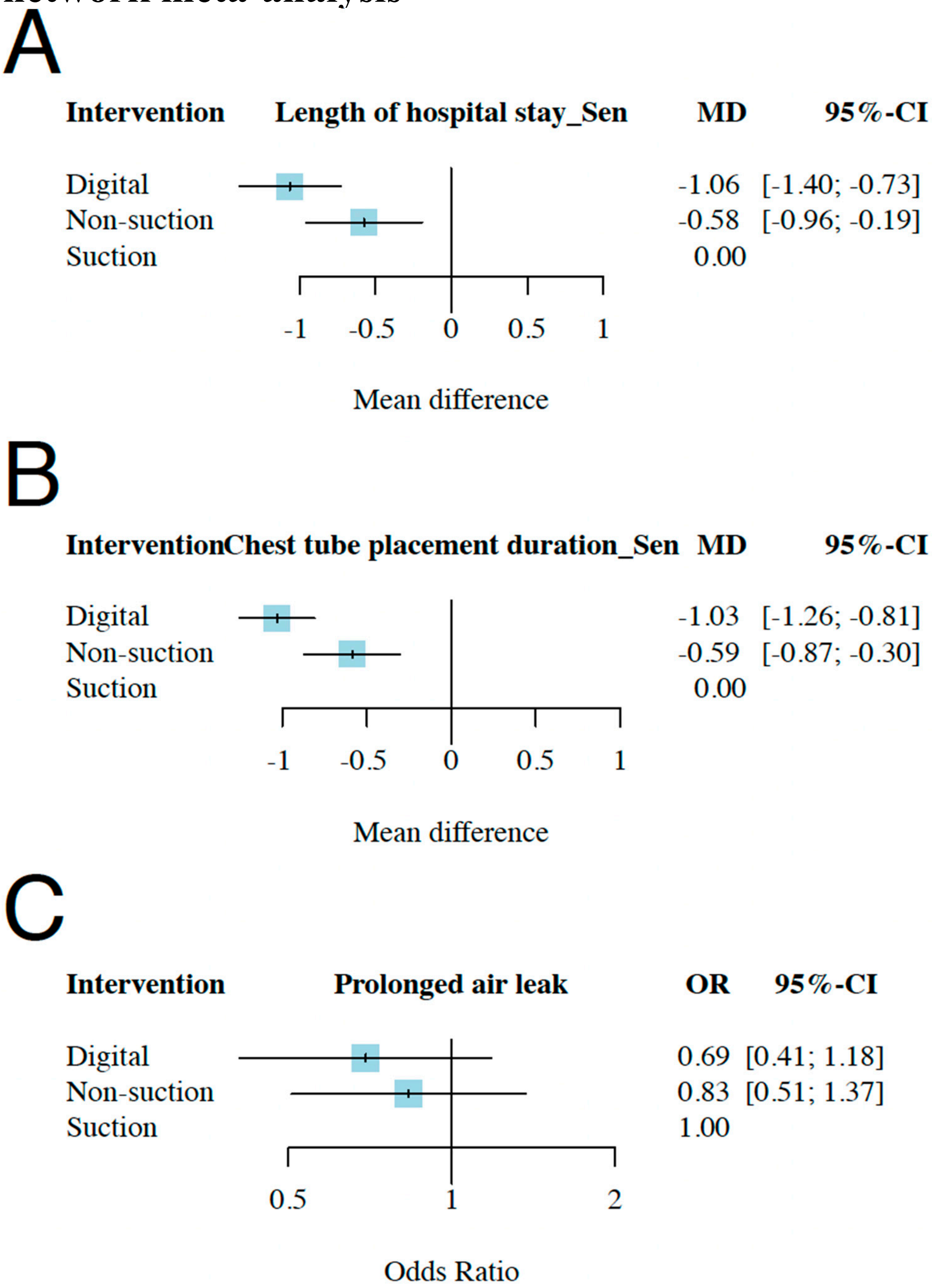

Figure S5. Sensitivity analyses for network meta-analysis of (A) the length of hospital stay, (B) the chest tube placement duration, as well as (C) the prolonged air leak.
